# Nerve Growth Factor and Pathogenesis of Leprosy: Review and Update

**DOI:** 10.3389/fimmu.2018.00939

**Published:** 2018-05-07

**Authors:** Tinara Leila de Souza Aarão, Jorge Rodrigues de Sousa, Aline Semblano Carreira Falcão, Luiz Fábio Magno Falcão, Juarez Antônio Simões Quaresma

**Affiliations:** ^1^Center of Health and Biological Sciences, State University of Para, Belem, Brazil; ^2^Tropical Medicine Center, Federal University of Para, Belem, Brazil; ^3^Evandro Chagas Institute, Ministry of Health, Ananindeua, Brazil

**Keywords:** nerve growth factor, leprosy, *Mycobacterium leprae*, pathogenesis, immunology

## Abstract

Neurotrophins are a family of proteins that regulate different aspects of biological development and neural function and are of great importance in neuroplasticity. This group of proteins has multiple functions in neuronal cells, as well as in other cellular populations. Nerve growth factor (NGF) is a neurotrophin that is endogenously produced during development and maturation by multiple cell types, including neurons, Schwann cells, oligodendrocytes, lymphocytes, mast cells, macrophages, keratinocytes, and fibroblasts. These cells produce proNGF, which is transformed by proteolytic cleavage into the biologically active NGF in the endoplasmic reticulum. The present review describes the role of NGF in the pathogenesis of leprosy and its correlations with different clinical forms of the disease and with the phenomena of regeneration and neural injury observed during infection. We discuss the involvement of NGF in the induction of neural damage and the pathophysiology of pain associated with peripheral neuropathy in leprosy. We also discuss the roles of immune factors in the evolution of this pathological process. Finally, we highlight avenues of investigation for future research to broaden our understanding of the role of NGF in the pathogenesis of leprosy. Our analysis of the literature indicates that NGF plays an important role in the evolution and outcome of *Mycobacterium leprae* infection. The findings described here highlight an important area of investigation, as leprosy is one of the main causes of infection in the peripheral nervous system.

## Introduction

**Figure 1:**
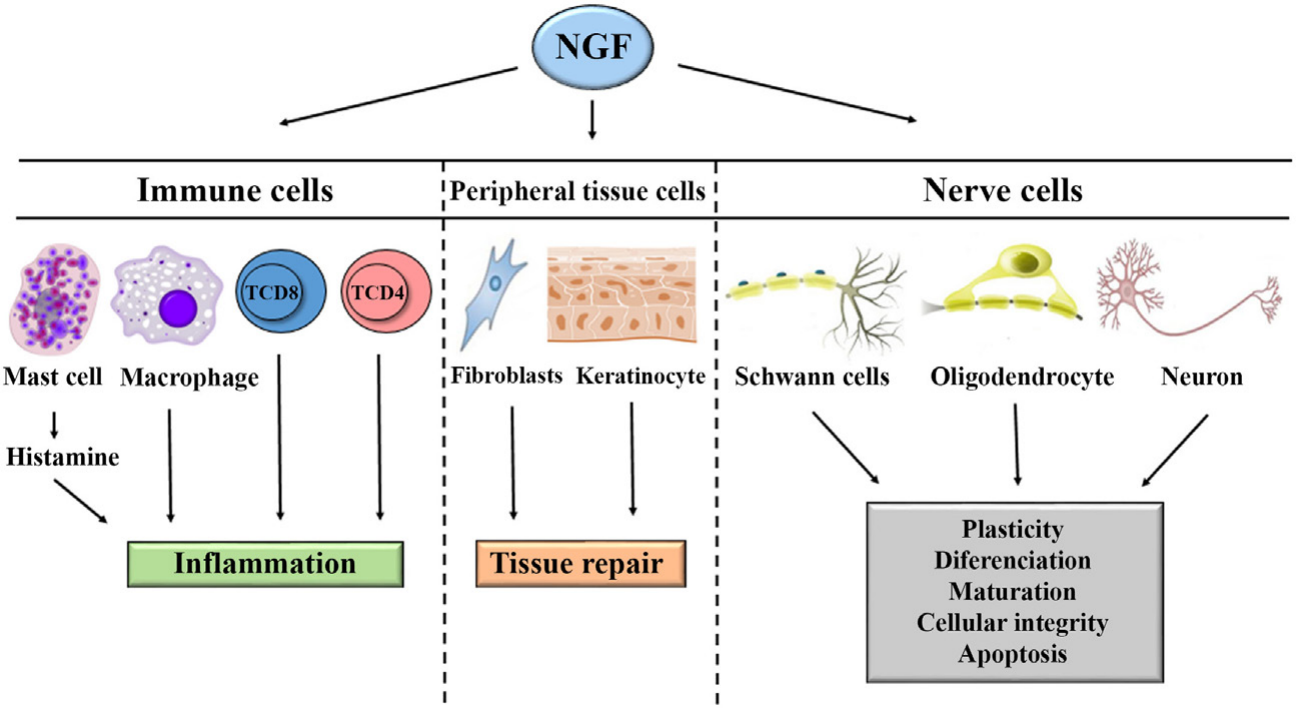
General mechanism of action of nerve growth factor (NGF) in the systemic immune response, neuroinflammation, regeneration, and tissue repair. NGF acts on lymphocytes, mast cells, and macrophages to induce inflammation. NGF acts on fibroblasts and keratinocytes to induce tissue repair. NGF acts on oligodendrocytes, Schwann cells, and neurons to induce repair or apoptosis.

**Figure 2:**
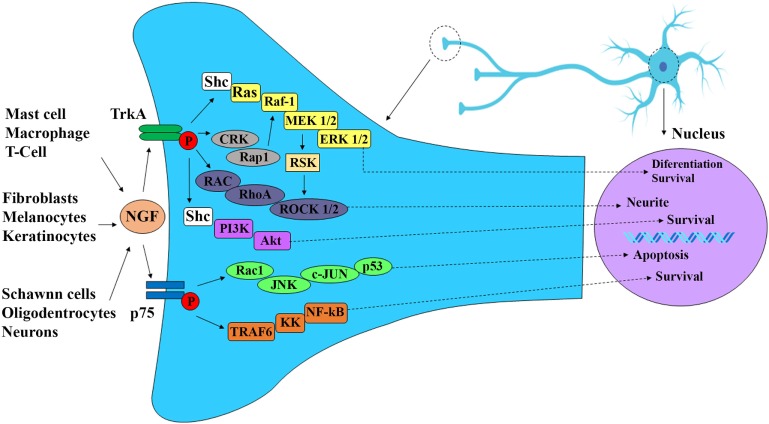
Nerve growth factor (NGF) signaling cascade. NGF is produced during development and into maturity by immune and nerve cells as well as peripheral effector cells, such as keratinocytes, melanocytes, and fibroblasts. NGF exhibits high affinity to receptor TrkA and low affinity to receptor p75. The binding of NGF through receptor TrkA induces autophosphorylation of TrkA receptor and activation of P13K/Akt pathway leading to neuronal differentiation, survival, and neurite. NGF binding to receptor p75 activate c-Jun kinase (JNK) and NF-κB pathways resulting in neuronal apoptosis and survival respectively.

The most extensively studied neurotrophin is nerve growth factor (NGF). This growth factor consists of 118 amino acids and has a molecular weight of 130 kDa. NGF was discovered as a growth factor that participates in the responses of sympathetic and sensory neurons regulating differentiation, neuronal regeneration, and the perception of pain (Figure 1) (1–5). In sensory neurons, NGF specifically binds to the TrkA receptor with high affinity and to receptor p75 with low affinity. The binding of NGF to receptor p75 seems to optimize the activity of TrkA (Figure 2) (6, 7). In skin and immune cells, NGF is produced by the proteolytic cleavage of its precursor, proNGF. proNGF is translocated to the lumen of the endoplasmic reticulum, transported through the exocytic pathway, and converted to its active mature biological form, NGF (3). The roles of NGF in physiological and pathological processes have been studied in several systems. Specifically, the significance of this molecule in responses to infectious and inflammatory diseases has recently been investigated in experimental models of Chagas disease, respiratory syncytial virus infection, herpes simplex virus infection, myalgic encephalomyelitis, and other diseases (8–15).

## Definition and Biological Aspects

Nerve growth factor was first described by Rita Levi-Montalcini (16), who showed its importance in the development, differentiation, maturation, and preservation of the integrity of sympathetic and sensory neurons (17). NGF is involved in modulating the sensitivity of peripheral nerve fibers to heat and pain in physiological and pathological events, such as genetic, metabolic (diabetes mellitus), and infectious neuropathies (18). Further supporting a relationship between NGF and leprosy, Scully and Otten and others by previous studies reported the involvement of NGF in sympathetic and sensory neuron apoptosis (Figures 1 and 2) (19–21).

Nerve growth factor is endogenously produced as proNGF during development and into maturity by immune and nerve cells, as well as peripheral effector cells, such as keratinocytes, melanocytes, smooth muscle cells, fibroblasts, and Schwann cells (5, 17, 18). It is also synthesized in other organs, such as the gonads, thyroid, parathyroid, and exocrine glands (e.g., salivary glands) (5, 21, 22). The expression and receptor binding affinity of NGF, as well as the duration and intensity of cellular events triggered by proNGF activation, determine its specific activity in effector cells or neurons (21, 23, 24).

Following its synthesis, NGF enters nerve endings by endocytosis, a process that permits NGF transport to the nerve cell body through contact with NGF receptors (NGF-R) located on the plasma membrane (25, 26). Several studies have described the roles of NGF in the central nervous system (CNS). In adults, NGF is involved in plasticity. However, the promotion of neuronal survival by NGF has only been observed in cholinergic neurons. NGF regulates the size of the cell body, dendritic branching, and neuronal connectivity (27, 28). In the peripheral nervous system, NGF acts on sympathetic and sensory neurons derived from the neural crest (29).

Nerve growth factor has been suggested to be involved in apoptosis of sympathetic and sensory neurons (20, 21). The binding of NGF to glial cells (i.e., oligodendrocytes and Schwann cells) through receptor p75 induces proapoptotic effects. However, these effects depend on NGF binding intensity and Trk receptor interactions (1, 30). Receptor p75 is a low-affinity accessory receptor for the TrkA receptor family to which all neurotrophins can bind. Receptor p75 seems to modulate the activity of TrkA and its intracellular signaling cascade. These receptors are present not only in nerve cells and CNS tumors, but also in immune cells such as macrophages, lymphocytes, and mast cells. These cells synthesize, store, and secrete proNGF, which suggests a possible role for NGF in the regulation of immune responses during inflammatory, infectious, and autoimmune processes (31, 32).

Thus, in addition to its primary functions in the nervous system, NGF participates in inflammatory processes and immune responses. NGF concentrations are increased during tissue inflammatory processes. Increased NGF levels can produce hyperalgesia through direct activation of nociceptors, which leads to CNS activation and neurogenic inflammation. Moreover, this process leads to local events comprising activation-induced release of histamine and increased numbers of mast cells and other immune system cells (7, 19).

Labouyrie et al. described the relationship between NGF and cells in normal and pathologic human lymphoid tissues and showed that NGF is involved in inflammatory or lymphoproliferative disorders (33). Immunoreactivity to the NGF-R (TrkA) has been observed in tissues, such as the thymic epithelium, cryptic tonsillar epithelium, epithelioid cells, multinucleated macrophages, and follicular dendritic cells. These findings demonstrate a relationship between NGF and the immune system. Owing to its production and activity in immune cells, NGF is thought by some to be a cytokine that participates in immune system events controlling the immune response in inflammatory and infectious processes (32, 34).

Different inflammatory and autoimmune diseases lead to altered expression of NGF. Increased anti-NGF antibody levels have been detected in patients with rheumatoid arthritis, systemic lupus erythematosus, and thyroiditis and are thought to contribute to the immune dysfunction and nerve damage observed in these diseases (21, 35, 36). However, the relationship between NGF autoantibodies and NGF expression remains unclear. Further studies are needed to investigate the effects of NGF autoantibodies on the expression of NGF and its receptors in autoimmune disease.

Other studies have shown an association between NGF and immune response mechanisms (37–42). For instance, Lambiase et al. have investigated the expression of NGF and the TrkA receptor in CD4+ T cells (38). Santambrogio et al. have shown that B cells express and secrete NGF, which in turn regulates the expression and secretion of calcitonin gene-related peptide by these cells *via* the TrkA receptor. This process has previously been described for sensory neurons (39). The immune response components interferon-β and interleukin (IL)-1β increase NGF-R expression and activate its signaling pathways in neurons and participate in the control of apoptosis in these cells (40). T and B lymphocytes, dendritic cells, and macrophages express NGF, TrkA, and the p75 neurotrophin receptor (Figure 2). In the context of innate immunity, NGF is related to the activation of IL-1β, nod-like receptor protein 1, NLRP3, and caspase-1 and contributes to inflammasome activation. TNF-α can induce differentiation and neuronal maturation through its interaction with NGF, which is in turn involved in the neuronal survival process (41, 42). Nevertheless, further investigation is needed to better elucidate the role of NGF in controlling the immune response in other cell types (41–43).

## NGF, Immune Response, and Leprosy

Leprosy is a chronic disease caused by *Mycobacterium leprae*, an intracellular bacillus that leads to loss of sensibility, innervation, intra-epidermal damage, and lesions associated with the loss of myelin in Schwann cells (44). Clinically, the different forms of the disease are characterized in part by the immune response patterns of the host (19, 44). According to the classification by Ridley and Jopling, leprosy has five clinical forms based on clinical, histopathological, immunological, and bacilloscopic criteria: borderline-tuberculoid, borderline-lepromatous, borderline-borderline, tuberculoid, and lepromatous (45). The initial stage of infection is referred to as the indeterminate form. The World Health Organization (WHO) classifies patients with leprosy as multibacillary or paucibacillary for treatment purposes. These classifications are made based on the identification of the bacilli and the number of lesions. Bacilloscopy is not always possible. Therefore, according to the WHO, the number of lesions can be used to classify patients into three groups: paucibacillary single-lesion leprosy (one skin lesion), paucibacillary leprosy (two to five skin lesions), and multibacillary leprosy (more than five skin lesions) (46).

In the tuberculoid form of the disease, the lesions are granulomatous and the individual displays an intense cell-mediated immune response (Th1) that prevents the proliferation of the bacillus. In the lepromatous form, the cell-mediated immune response is characterized by an anti-inflammatory cytokine profile (Th2) that contribute to multiplication of the bacillus in macrophage phagosomes. In the borderline form, the patients exhibit immunological and histopathological characteristics that vary between those of the tuberculoid and lepromatous forms (47).

Nerve injury-associated tissue damage is arguably the most important clinical consequence of leprosy (47). In the process of leprosy-associated neuropathy, the presence of bacilli in nerve endings and Schwann cells induces a response mediated by macrophages and other cells that eventually leads to the appearance of immune-mediated lesions. In this process, cytokines, such as TNF-α, IL-6, and IL-17, may contribute to the evolution of neural lesions and deformities that are characteristic of some forms of the disease. The immune response and inflammation are not only defined by the presence of chemical mediators, such as cytokines and chemokines. Rather, inflammatory edema is also very important in the neuropathy associated with leprosy, which can induce degeneration of neural fibers. There are strong positive correlations among the levels of NGF, NGF-R, and TGF-β in patients with leprosy. This indicates that the above factors have synergistic actions that reduce tissue damage resulting from nerve injury (48). Study by Antunes et al. (48) in patients with the neuritic form of leprosy (pure neural form or primary neural form), it was observed that the NGF-R immunoexpression was lower in nerve fibers and Schwann cells when compared to normal controls. In this study, hypoesthesia was correlated with decreased expression of NGF-R and protein gene product (PGP) 9.5, and electroencephalographic changes were observed in patients with altered immunolabeling for neurofilaments and PGP 9.5. These data point to a key role of NGF in the pathogenesis of neural lesions in leprosy.

Leprosy is the most common cause of non-traumatic neuropathy and is a classic example of an infectious neurodegenerative disease of the peripheral nervous system. It is estimated that more than one-fourth of patients with leprosy have some degree of disability and that about half of these patients have grade 2 disability corresponding to permanent neurological damage (44–59).

Studies have shown that different levels of NGF are associated with lepromatous and tuberculoid leprosy such that higher levels of NGF are associated with lepromatous forms and low levels of NGF are associated with tuberculoid forms of the disease (60). Specifically, Facer et al. have demonstrated the importance of NGF in leprosy (44). In patients with leprosy with and without lesions, TrkA receptors were shown to be present in subepidermal fibers and TrkA receptor messenger RNA was produced in the skin. The authors of the above study also evaluated the integrity of nerve endings and found that the presence of NGF in keratinocytes was correlated with deficient thermal sensation. Anand et al. detected low NGF levels in nerve and skin lesions of patients with leprosy and demonstrated that these low NGF levels contributed to the loss of NGF-dependent nociceptive fibers in damaged skin (19). Another study by Anand demonstrated that the loss of interaction between keratinocytes and nerves in affected skin drastically reduced the flow of NGF (61). Schwann cells produce NGF in response to axonal degeneration. However, while the levels of NGF are sharply reduced in the affected nerve trunks in patients with neuropathic lesions, there is a local increase in NGF levels in patients with chronic cutaneous hyperalgesia (61). The use of anti-NGF antibodies may be effective in treating hyperalgesia in patients with neuropathy and compromised nerve endings. In addition, physiological combinations of NGF, NT-3, and glial cell line-derived neurotrophic factor may assist in the reestablishment of homeostasis, and may thus be used in the treatment of neuropathic pain (61).

Other studies have suggested that NGF may restore pain sensitivity. Thus, NGF could play an important role in the prevention of ulcerations resulting from nociception loss, as observed in leprosy and other peripheral neuropathies (50, 61). Some reports indicate that anti-NGF treatment may promote analgesia in patients with hyperalgesia, which suggests a modulatory role for NGF in nociception (61).

**Figure 3:**
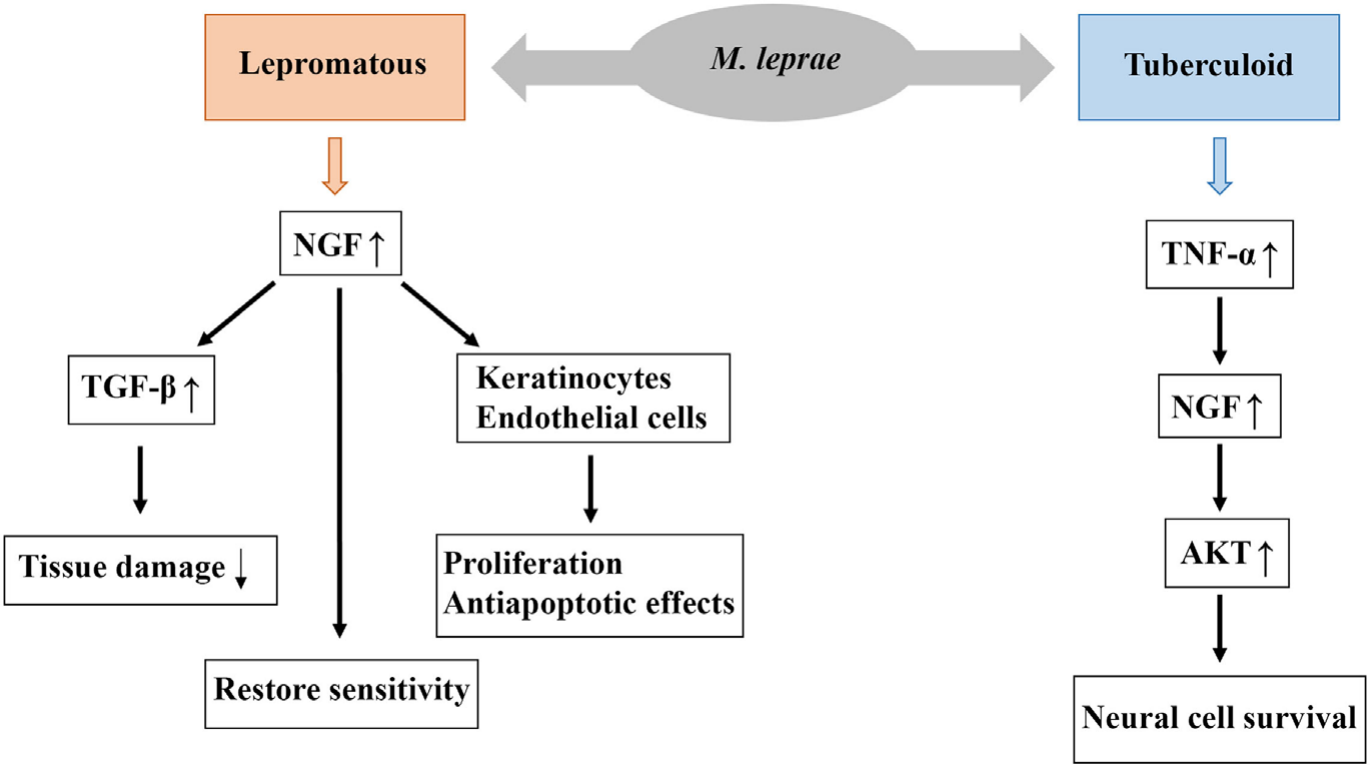
Possible biological role of nerve growth factor (NGF) in the pathogenesis of leprosy. Higher levels of NGF are associated with lepromatous forms, and this increased NGF expression stimulates the expression of TGF-β, which reduce tissue damage resulting from nerve injury. Moreover, NGF restore sensitivity and exerts proliferative and antiapoptotic effects on keratinocytes and endothelial cells. Low levels of NGF are associated with tuberculoid forms and this low expression of NGF stimulates the expression of TNF-α which may contribute to the evolution of neural lesions. Low levels of NGF may also contribute to the development of neuropathy, such as sensitivity loss, nerve demyelination, and degeneration.

Immunostaining of damaged tissues using anti-NGF and anti-NGF-R antibodies revealed higher expression of NGF in patients with lepromatous leprosy and lower expression of NGF in those with the indeterminate form of the disease (49). In lepromatous forms of leprosy, higher mean expression levels of NGF and its receptor are associated with larger and more diffuse lesion patterns and greater nerve involvement. The presence of NGF is generally more apparent in patients with the highest probability of nerve damage (i.e., those with lepromatous leprosy and young patients) (Figure 3) (52, 53).

Several important findings have elucidated the role of NGF as an immune response mediator (38). In addition to its well-documented involvement in the differentiation and growth of neurons, there is growing evidence that NGF exerts a broad spectrum of effects on immune cells. As a result, NGF is considered a pleiotropic molecule involved in different functions (i.e., neuropeptide modulation and tissue healing) that influence neural development, immune function, and injury responses (54).

Imbalance in the proNGF/NGF ratio, increased expression levels of TNF-α and p75 neurotrophin receptor, and impaired TrkA phosphorylation have been reported in microvascular preparations from Cre-proNGF transgenic mice when compared to normal control animals (43). TNF-α can induce differentiation and neuronal maturation through its interaction with NGF, which is in turn involved in the neuronal survival process (62). Endogenous TNF-α expression induced by NGF leads to a positive feedback loop comprising Akt activation through TNF receptor 2. This pathway promotes the survival of normal neural cells. Some studies suggest that TNF-α and IL-1β control the activity of NGF in human synovial fibroblasts modulating the physiopathology of osteoarthritis (41, 62).

The relationship between NGF and TGF-β in glial cells has been described in a study on mice and rats with spinal injury (37). Following injury, TGF-β1 levels increased, which in turn led to NGF messenger RNA and protein expression in the glial cells of these animals. In leprosy, the main target of *M. leprae* is the Schwann cell, which is the predominant cell type in the peripheral nervous system. Degradation of Schwann cells by *M. leprae* favors the development of peripheral neuropathy, which is the leading cause of morbidity in patients with leprosy. NGF may act as a protective factor for Schwann cells, and low levels of NGF may contribute to the development of neuropathy (38, 39).

Studies of systemic diseases (i.e., diabetes mellitus and osteoarthritis) have revealed an interaction between NGF and TGF-β (62, 63). Neurotrophins play a crucial role in the differentiation and survival of nerve cells. The characteristic positive correlations among NGF, NGF-R, and TGF-β in the clinical forms of leprosy highlight the interdependence of these factors (49).

Studies of lesions of patients with borderline leprosy have revealed strong correlations between TGF-β and NGF, and TGF-β and NGF-R (*r* = 0.8722 and *r* = 0.7257, respectively), with highly significant *p*-values for the two correlations (*p* < 0.0001 and *p* = 0.0015, respectively) (48, 49). The borderline form of leprosy is immunologically dynamic and oscillates between the two polar forms. In patients with borderline-tuberculoid, borderline, and borderline-lepromatous leprosy, the progressive reduction in the cell-mediated response from the borderline-tuberculoid to the borderline-lepromatous form is accompanied by more extensive skin lesions and nerve damage, as well as increased bacillary burden and antibody levels (63, 64). In borderline forms of the disease, neurological manifestations resulting from immunological instability are characterized by nerve trunk impairment and frequent reactional episodes, which lead to early and asymmetrical nerve injuries and physical disability. This process is caused by increased numbers of bacilli in nerve branches close to Schwann cells (49). Cunha analyzed the relationships between the clinical forms of leprosy and episodes of neuritis. They found that patients with the borderline form of leprosy are 2.69 times more likely to progress to neuritis than those with the lepromatous form of the disease (65, 66).

During healing, NGF activates processes involved in the restoration of innervation (65, 67). In addition to inducing fibroblast migration, NGF exerts proliferative and antiapoptotic effects on keratinocytes and endothelial cells (68, 69). Moreover, NGF may be important for potentiating injury-specific responses through proinflammatory effects on neutrophils, eosinophils, mast cells, and T lymphocytes (67). The interactions of NGF in the tissue microenvironment are complex, and its relation to TNF-α, which can induce apoptosis in Schwann cells by binding to specific death receptors, may lead to antagonistic effects. This is because NGF can activate survival signals in the target cell. In fact, the same cytokine can have antagonistic effects depending on its interactions with specific receptors and the intracellular cascade activated after the activation of these receptors (Figure 3) (41–43, 62).

Cell-mediated immune responses may be beneficial against bacterial infections; however, inflammation can lead to irreversible tissue damage. Nerve injury occurs in approximately 10% of patients with paucibacillary leprosy and 40% of patients with multibacillary leprosy and is particularly acute in patients with reverse reactions (69, 70).

TGF-β participates in the tissue repair process as an anti-inflammatory agent during intense inflammation by inducing nerve and tissue regeneration (67, 71). Higher TGF-β expression in patients with the lepromatous form of the disease is associated with a higher frequency of apoptosis in lesions, especially within Schwann cells (72). The positive correlation between TGF-β expression and NGF expression may be associated with the protection and regeneration of nerve endings (60).

In lepromatous leprosy, neurological manifestations progress slowly over many years and involve small nerve branches and multiple nerve trunks within the skin (73, 74). Peripheral nerve injury, or any pathological condition that causes an interruption between the target organ and the nerve cell body, acts as a signal that induces non-neural cell populations (e.g., fibroblasts) to produce NGF. The induction of NGF synthesis in these cells is also modulated by cytokines, which invade the site of nerve injury where nerve regeneration is initiated (74).

The expression of p75 receptor in peripheral nerve cells is induced by loss of contact between the target organ and the axon (60). Some studies suggest that the p75 receptor is involved in axonal NGF uptake, reflecting the importance of NGF in efficient nerve regeneration. Patients with lepromatous leprosy have nerve trunk lesions, as well as multiple mononeuropathies and polyneuropathies (33, 71–76). The relationship between NGF and TGF-β is a key determinant of the actions of NGF in patients with the lepromatous form of the disease. Coordination between NGF and TGF-β responses in inflammatory processes following tissue damage is thus fundamentally important in remodeling and tissue repair (Figure 3) (60).

## Need for Future Research

Prospective cohort studies and intervention studies evaluating patients with different clinical forms of the disease and different reactive states (erythema nodosum and reverse reaction types) may help better elucidate the relationship between NGF and the immune response and the factors contributing to the protection and regeneration of nerves affected during infection. Further analysis of the relationship among tissue levels of NGF and a large panel of pro- and anti-inflammatory cytokines, blood levels of NGF, and the immune response is important for a better understanding of the involvement of NGF in the pathophysiology of chronic granulomatous peripheral nerve infections, especially leprosy.

## Conclusion

This review demonstrates that, within lesions associated with leprosy, NGF and TGF-β respond to inflammatory processes and tissue damage while triggering tissue remodeling. Further studies are needed to elucidate the broad role of NGF in the pathogenesis of leprosy. Although our understanding of the effects of NGF on nerve damage has increased, further insights into the functional roles of NGF (and other neurotrophins) in normal skin and during disease progression are needed.

## Author Contributions

TA, JS, AF, LF, and JQ conceived and wrote the manuscript.

## Conflict of Interest Statement

The authors declare that the research was conducted in the absence of any commercial or financial relationships that could be construed as a potential conflict of interest.
